# Newly designed 16S rRNA metabarcoding primers amplify diverse and novel archaeal taxa from the environment

**DOI:** 10.1111/1758-2229.12684

**Published:** 2018-09-12

**Authors:** Mohammad Bahram, Sten Anslan, Falk Hildebrand, Peer Bork, Leho Tedersoo

**Affiliations:** ^1^ Department of Botany Institute of Ecology and Earth Sciences, University of Tartu 40 Lai St, Tartu; ^2^ Department of Organismal Biology Evolutionary Biology Centre Uppsala University Norbyvägen 18D, Uppsala Sweden; ^3^ Department of Ecology Swedish University of Agricultural Sciences Ulls väg 16, 756 51 Uppsala Sweden; ^4^ Zoological Institute Braunschweig University of Technology Mendelssohnstr. 4, 38106 Brunswick Germany; ^5^ Structural and Computational Biology European Molecular Biology Laboratory Heidelberg Germany; ^6^ Max Delbrück Centre for Molecular Medicine Berlin Germany; ^7^ Department of Bioinformatics, Biocenter University of Würzburg Würzburg Germany; ^8^ Natural History Museum University of Tartu 14A Ravila, 50411 Tartu Estonia

## Abstract

High‐throughput studies of microbial communities suggest that Archaea are a widespread component of microbial diversity in various ecosystems. However, proper quantification of archaeal diversity and community ecology remains limited, as sequence coverage of Archaea is usually low owing to the inability of available prokaryotic primers to efficiently amplify archaeal compared to bacterial rRNA genes. To improve identification and quantification of Archaea, we designed and validated the utility of several primer pairs to efficiently amplify archaeal 16S rRNA genes based on up‐to‐date reference genes. We demonstrate that several of these primer pairs amplify phylogenetically diverse Archaea with high sequencing coverage, outperforming commonly used primers. Based on comparing the resulting long 16S rRNA gene fragments with public databases from all habitats, we found several novel family‐ to phylum‐level archaeal taxa from topsoil and surface water. Our results suggest that archaeal diversity has been largely overlooked due to the limitations of available primers, and that improved primer pairs enable to estimate archaeal diversity more accurately.

## Introduction

Recent 16S rRNA metabarcoding and metagenomics analyses suggest that Archaea are a widespread prokaryotic group that plays important roles in carbon and nitrogen cycling, particularly in key biogeochemical processes such as methanogenesis and nitrification (Offre *et al*., 2013). Yet, the diversity and importance of Archaea in various environments remains poorly understood (Adam *et al*., 2017).

Ribosomal RNA gene‐based metabarcoding is the most widely used identification method in microbiology that has led to the discovery of enormous diversity of currently uncultured microbes (Spang *et al*., 2015). Although several primers are available for amplification of bacterial and archaeal 16S rRNA genes, these fail to amplify a broad spectrum of archaeal lineages (Narasingarao *et al*., 2012; Eloe‐Fadrosh *et al*., 2016). Compared to Bacteria, Archaea often represent a small fraction in most prokaryote metabarcoding data sets. The relatively low abundance of Archaea in metabarcoding data sets (Bahram *et al*., 2018) may at least partly stem from their mismatches to commonly used prokaryotic PCR primers (Baker *et al*. 2003; Gantner *et al*. 2011). A single primer‐template mismatch may render many taxa undetected (Bru *et al*., 2008). In addition, the highly divergent 16S rRNA gene in various groups of Archaea (Baker *et al*., 2003) makes it difficult to design universal primers for prokaryotes (Parada *et al*., 2016) and primers specifically targeting Archaea (Baker *et al*., 2003). A number of attempts have proposed general and phylum specific primers for prokaryotes *in silico* (Wang and Qian, 2009; Klindworth *et al*., 2013), but only few actual primer testing experiments have been performed for Archaea, inasmuch as primer performance may differ between *in silico* and wet‐lab experiments (Raymann *et al*., 2017). Furthermore, most of these primers were designed when sequence databases lacked important archaeal phylum‐level groups such as ‘DPANN’ and Lokiarchaeota (Rinke *et al*., 2013; Eloe‐Fadrosh *et al*., 2016; Raymann *et al*., 2017).

Although the V1 and V2 variable subregions of the 16S rRNA gene provide the greatest resolution among Archaea taxa (Hartmann *et al*., 2010), much of the primer development has focused on the less variable V4 and/or V5 regions, which is a popular target for metabarcoding of Bacteria (Caporaso *et al*., 2012; Walters *et al*. 2016; Bahram *et al*., 2018). Furthermore, much of the primer development for prokaryotes and particularly for Archaea has been focused on short fragments suitable for second‐generation sequencing technologies such as Illumina and Ion Torrent. Development of third‐generation sequencing methods such as Pacific Biosciences and Oxford Nanopore enables to recover the entire 16S marker that enables better separation among taxa and a higher resolved phylogenetic placement (Schloss *et al*., 2016; Tedersoo *et al*., 2018). Here we designed nine novel degenerate primers, targeting various 16S subregions ranging from 250 to 1500 bp. We tested the performance of 27 Archaea‐specific and ‘universal’ primer pairs for amplification as well as third‐generation sequencing of Archaea. Our ultimate objective is to provide recommendations of primer choice for researchers specifically targeting Archaea in various environments using any high‐throughput sequencing platform.

## Materials and methods

### *Sampling and molecular analysis*

Sampling design followed a standard protocol described in Tedersoo and collegues (2014) in which 40 subsamples (5 cm diam. to 5 cm depth) of topsoil or surface water were collected from 0.25‐ha study area and pooled for DNA extraction and chemical analysis. For this study, we selected eight composite soil and mangrove samples from various continents and biomes, including moist tropical forests (swamp), moist subtropical forests, Mediterranean, dry tropical forests (riparian), moist tropical forests, flooded grassland, and a soda lake (Supporting Information Table S1).

**Figure 1:**
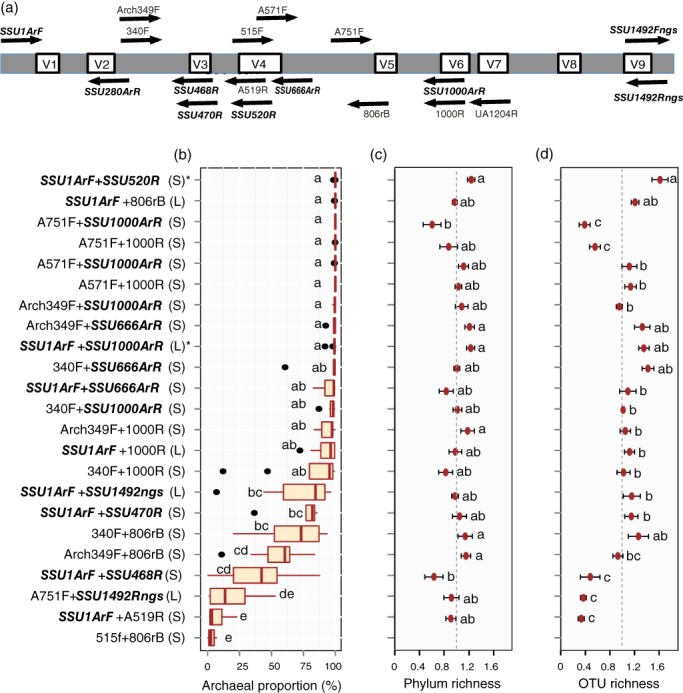
Details and relative performance of primers used in this study for identification of Archaea. A. Map of small ribosomal RNA subunit (SSU) and primers used in this study. Primers designed in this study are indicated in italic. B–D. The performance of tested primer pairs (sorted by the proportion of Archaea sequences) for amplification of archaeal 16S rRNA genes: (B) the proportion of Archaea sequences across samples, (C) relative OTU richness in relation to average richness in each sample, and (D) relative phylum richness in relation to average richness in each sample. The solid line indicated average level among samples. Different letters denote significant differences (*P* < 0.05). Error bars show standard error. Note that several primer pairs were excluded from **B–D** due to their performance in amplifying Archaea. For the same reason, the reference primer pair 515f + 806rB was excluded from C and D. S, short amplicons (< 700 bp); L, long amplicons (> 700 bp); * recommended based on this study.

To design novel Archaea‐specific primers, we used the alignment of the SILVA sequence database version 128 (Quast *et al*., 2012) including a single representative sequence for entities clustered at 90.0% and 85% sequence identity levels for Archaea and Bacteria respectively. The reference alignment contained 1901 and 8647 sequences of Archaea and Bacteria respectively. By visual inspection of the Archaea part of the alignment, we selected all potential 16S rRNA gene stretches for potential primer design using the following criteria: (i) length > 17 bp; (ii) < 25% mismatches to Archaea consensus in any representative sequence. This resulted in 21 sequence stretches, which were used along with sequences of all taxa for degenerate primer development based on another set of criteria: (iii) no terminal (two 3′ nucleotides) mismatches to 99.5% Archaea sequences; (iv) < 2 mismatches to 99.5% of Archaea sequences; (v) none of the rare Archaea phyla (others than Euryarchaeota and Crenarchaeota) may be excluded within the 0.5% mismatched groups; (vi) terminal mismatch(es) or > 1 non‐terminal mismatch to > 99.5% non‐Archaea sequences; (vii) AT/GC ratio of 40%–60%; (viii) *T*_m_ 56–62 °C; (ix) <50% degenerate positions. Whenever feasible, we considered both forward and reverse primers. Since very few primers provided high coverage for Archaea and no coverage for other groups, we also considered primers that did not fully exclude other groups, but restricted their use to combine only with fully Archaea‐specific primers. These relatively stringent criteria resulted in the development of four and five primers specific to Archaea and Archaea + some groups of Bacteria respectively (Table 1; Fig. 1A). As a reference, we also chose the universal primer pair 515F + 806rB (Caporaso *et al*., 2012) used by several global microbiome projects and several widely used or previously recommended primers (Supporting Information Table S1) for comparison. We combined the primers in 27 pairs to cover amplicons from 250 to 1500 bp.

**Table 1:** Primers designed and/or tested in this study.

Primer	Position	Sequence	Orientation	Target group	Reference
SSU1ArF	1	TCCGGTTGATCCYGCBRG	fwd	Archaea	This study
SSU280ArR	280	TCAGWNYCCNWCTCSRGG	rev	Archaea	This study
SSU666ArR	666	HGCYTTCGCCACHGGTRG	rev	Archaea	This study
SSU1000ArR	1000	GGCCATGCAMYWCCTCTC	rev	Archaea	This study
SSU520R	520	GCTACGRRYGYTTTARRC	rev	Prokaryotes	This study
SSU470R	470	DCNGCNGGTDTTACCGCG	rev	Prokaryotes	This study
SSU468R	468	GNDCNGCNGGTDTTACCG	rev	Prokaryotes	This study
SSU1492Rngs	1492	CGGNTACCTTGTKACGAC	rev	Prokaryotes	This study
SSU1492Fngs*	1492	GTCGTMACAAGGTANCCG	fwd	Prokaryotes	This study
1000R	1000	GGCCATGCACYWCYTCTC	rev	Archaea	Gantner *et al*. (2011)
UA1204R	1204	TTMGGGGCATRCIKACCT	rev	Archaea	Baker *et al*. (2003)
340F	340	CCCTAYGGGGYGCASCAG	fwd	Archaea	Gantner *et al*. (2011)
A571F	571	GCYTAAAGSRNCCGTAGC	fwd	Archaea	Baker *et al*. (2003)
A751F	751	CCGACGGTGAGRGRYGAA	fwd	Archaea	Baker *et al*. (2003)
A519R/S‐D‐Arch‐0519‐a‐A‐19	519	GGTDTTACCGCGGCKGCTG	rev	Archaea	Wang & Qian. (2009)
Arch349F/S‐D‐Arch‐0349‐a‐S‐17	349	GYGCASCAGKCGMGAAW	fwd	Archaea	Takai & Horikoshi (2000)
515F	515	GTGCCAGCMGCCGCGGTAA	fwd	Universal	Turner *et al*. (1999)
806rB	806	GGACTACNVGGGTWTCTAAT	rev	Universal	Apprill *et al*. (2015)

* Not tested.

One of the primers were supplemented with a 10–11‐base identifier tag (Tedersoo *et al*., 2014). PCR reactions contained 0.6 μl DNA extract, 0.5 μl of primers (20 pmol), 5 μl 5xHOT FIREPol Blend Master Mix (Solis Biodyne, Tartu, Estonia) and 13.4 μl double‐distilled water. PCR conditions included an initial 15 min at 95 °C, followed by 30 cycles at 95 °C for 30 s, 55 °C for 30 s, 72 °C for 1 min, and a final cycle of 10 min at 72 °C using Phusion polymerase (Supporting Information Table S2). For each sample, two replicate PCR products were pooled and their relative quantity was estimated by running 5 μl amplicon DNA on 1% agarose gel for 15 min. DNA samples that failed to yield a visible band or yielded a very strong band were re‐amplified using 35 and 25 cycles respectively. Purification of short amplicons was done using FavorPrep Gel/PCR Purification kit (Favorgen Biotech Corp., Vienna, Austria). Long amplicons were purified with Agencourt AMPure XP magnetic beads (Beckman Coulter Genomics, Danvers, MA, USA). Library preparation was performed as described previously (Tedersoo *et al*., 2018). For equimolar pooling of samples, approximate number of fragments was calculated for each library, followed by pooling of the same amount of fragments from each library. The final products were sequenced on PacBio Sequel instrument in the Norwegian Sequencing Centre (University of Oslo). All consensus sequences were submitted to the Short Read archive (SRA) under Accession No. SRP148434.

### *Bioinformatics and statistical analyses*

Bioinformatics were performed in the PipeCraft platform v1.0 (Anslan *et al*., 2017). First, PacBio circular consensus sequences (≥ 2 passes) were quality‐filtered using vsearch (Rognes *et al*., 2016) with the following settings: ‐‐fastq_maxee 1 ‐‐fastq_minlen 150 ‐‐fastq_maxns 0. The quality‐filtered reads were demultiplexed using mothur with the following settings: bdiffs = 1; pdiffs = 2 (Schloss *et al*., 2009). All resulting reads were clustered at 97% sequence identity threshold and chimeric sequences were removed using Uparse (Edgar, 2013). The taxonomic annotations of representative sequences (most abundant) per OTU were performed using the Naïve Bayesian classifier in mothur, with SILVA 132 database (Ref) as a reference database.

To test the performance of our primers in amplifying previously undetected archaeal lineages in amplicon data sets, we compared the representative sequences of OTUs uncovered by our primers with those from metagenomics and amplicon reference data sets. Using ‘usearch_global’ command of vsearch, we matched our sequences with 16S rRNA sequences of SILVA as well as a recently published metagenomic data set derived from various environments (Karst *et al*., 2018), abbreviated MG in the following. We further aligned our sequences with the closest representative sequences from SILVA and MG (at 90% identity threshold) to generate a phylogenetic tree. Sequences were aligned against SILVA seed using mothur and manually edited. For this analysis, only long representative sequences (> 450 bp) of archaeal OTUs from the forward primer SS1Arf were used. Maximum likelihood phylogenetic analyses were performed using RAxMLVersion 8 (Stamatakis, 2016). New Archaea taxa were determined based on the sequence identities of OTUs with those from SILVA and MG (species, genera, families, orders, classes and phyla at 97%, 95%, 92%, 89%, 86% and 83% respectively) following ref. (Konstantinidis *et al*., 2017) and using Blastn search. The tree was visualized and annotated using ITOL (Letunic and Bork, 2016).

To test differences in the proportion of Archaea sequences, number of OTUs and number of phyla recovered, we performed univariate analyses using Statistica 13.3 software (StatSoft, Inc., Tulsa, Ok). Due to different number of recovered sequences, square‐root of sequencing depth was used as a covariate. Composite samples were treated as blocks to reduce the error term. Tukey's tests were performed to test for significant differences among the primer pairs. in capturing Archaea diversity. Relative effects of primer pairs on taxonomic composition (Hellinger distance) were tested using Permanova+ (Anderson *et al*., 2008) and presented as nonmetric multidimensional scaling (NMDS) ordination plots. Rarefaction analyses were performed in Vegan package (Oksanen *et al*., 2007) of R version 3.0.3. The performance of the primer pairs was also evaluated *in silico* using TestPrime 0.1 of SILVA (Klindworth *et al*., 2013).

## Results and discussion

Of the 27 tested primer pairs, four (A571F + UA1204R, A751F + UA1204R, A571F + SSU1492Rngs, SSU1ArF + UA1204R) yielded no PCR products or a smear on the gel. The commonly used universal prokaryote primers 515F + 806rB (Caporaso *et al*., 2012) performed worst for analysis of Archaea by producing only 2.1% of Archaea sequences on average and covering only Euryarchaeota and Thaumarchaeota phyla (Fig. 1B–D, Supporting Information Fig. S2 and Table S3). This indicates that the diversity of Archaea has been largely underestimated in studies utilizing universal primers, despite that 515F + 806rB has been modified to enhance Archaea read abundance (Caporaso *et al*., 2012; Parada *et al*., 2016). The *in silico* analysis revealed that the newly designed primer pairs outperformed the current specific primers in terms of specificity and coverage (Supporting Information Table S4). Therefore, we statistically compared the performance of primers intended to be more or less specific to Archaea. At tested conditions, primer combinations including primers SSU1ArF, SSU520R, 340F, SSU666ArR and SSU1000ArR yielded the largest number of OTUs when accounting for differences in sequencing depth (Supporting Information Fig. S1). These primers cover the V1 and/or V2 regions that are considered the most variable in 16S rRNA gene (Hartmann *et al*., 2010), which may enhance the recovered richness. However, these primers are also able to recover relatively high phylum richness and high proportion of Archaea sequences. The accumulation curves of Archaea diversity with increasing sequencing depth approximately approached asymptote for these primers, in strong contrast to 515F + 806rB (Supporting Information Fig. S1). Conversely, combinations including primers A519R, A751F and SSU468R yielded low OTU diversity, relatively low phylum‐level richness and low Archaea proportion. Of individual primer pairs, SSU1ArF + SSU520R performed best in all three aspects, but none of the samples yielded members of Altiarchaeota, Lokiarchaeota or Parvarchaeota. These three rare groups were recovered from < 50% of the samples and using < 33% of primer pairs, indicating potential issues with detection limit. Primer pairs 340F + 806rB and Arch349F + 806rB recovered nine of the 12 major groups of Archaea present in databases, indicating high affinity to Archaea of the 806rB primer. In some combinations, the primers SSU1ArF, 806rB and SSU1492Rngs yielded a large proportion of bacterial, eukaryote or metagenomics sequences (Supporting Information Fig. S2; Supporting Information Table S2).

For short amplicons, we recommend primer pairs SSU1ArF + SSU520R and 340F‐806rB to sequence the V1/V2 and V4/V5 SSU regions of SSU using Illumina or Ion Torrent platforms. For longer amplicons, the SSU1ArF + SSU1000ArR primer pair is promising. Although the SSU1ArF + SSU1492Rngs yields relatively high richness, it recovered 12.1% non‐target sequences and was sometimes difficult to amplify. In all combinations, the SSU1492Rngs primer failed to amplify the common phylum Bathyarchaeota, and the reads yielded conspicuously lower Thaumarchaeota to Euryarchaeota ratio compared with other primer combinations. Primer pairs explained 3.2% of phylum level distribution across samples (*F*_2,169_ = 7.51; *P* < 0.05; not shown).

A comparison of archaeal lineages uncovered by our primers with those from metagenomics and amplicon sequencing data sets from various environments revealed several novel Archaea taxa from the genus to the phylum levels (Supporting Information Fig. S3 and Table S3). This analysis revealed that our sequences better matched those in PCR‐free than PCR based data set, i.e., MG and SILVA data sets respectively (at 97% similarity threshold: 19.1% vs. 36.2%; at 90% similarity threshold: 82.5% vs. 47.5% respectively). In addition, the composition of archaeal phyla recovered by our primers was more similar to MG than SILVA data set (Supporting Information Table S5), indicating low dependence of the primer performance on the database used (i.e., SILVA).

**Figure 2:**
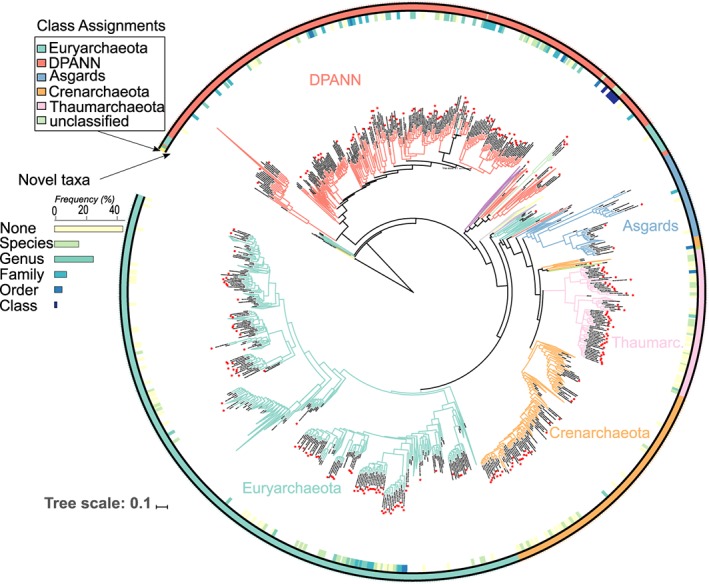
Maximum likelihood phylogenetic tree constructed from alignment of representative sequences of archaeal OTUs recovered from SSU1 primers (> 500 bp) along with their closest matches (90% identity) in SILVA and MG reference databases. Novel taxa are marked. The outer circle represents the colour‐coded class as determined by RDP. The inner circle shows the similarity to the closest hit of a sequence in SILVA or MG database, with the colour indicating the thresholds used to delineate new taxa (see Supporting Information Table S5). Archaeal sequences generated in this study are indicated by red stars at the tips of the phylogeny. For an interactive version of this figure including bootstrap values, see https://itol.embl.de/tree/213661668247441526333299#. The histogram shows the proportion of novel archaeal taxa (species, genera, families, orders and classes at 97%, 95%, 92%, 89% and 86% respectively; (Konstantinidis *et al*., 2017)), uncovered in this study. For more details, see Supporting Information Table S4.

Several clades previously known only from genomics and metagenomics data were amplified by our newly designed primers, in particular Lokiarchaeia and Odinarchaeia from Asgards (Spang *et al*., 2015) (details and sequences are given in Supporting Information Table S6). Despite this, Asgards remained rare in our data, perhaps owing to the low coverage of our primers for this group (Supporting Information Table S4). Certain members of the recently proposed superphylum DPANN (Rinke *et al*., 2013), were among the top most abundant Archaea phyla in terms of sequence abundance in our sequence data set. Many members of these phyla in our data set had only little similarity to SILVA or MG databases (Fig. 2; Supporting Information Table S5 and S6). A phylogenetic tree based on our and the closest SILVA/MG sequences shows that these groups are largely unexplored, representing new species, genera, families, orders, classes or perhaps even phyla (Fig. 2). Several OTUs belonging to Woesearchaeota (formerly DHVEG‐6) appeared to have only a few closely related matches in SILVA and metagenomics data sets (Fig. 2). This phylum is one of the recently introduced and deep branching Archaeal lineages belonging to DPANN (Adam *et al*., 2017). We also note that two clades within Crenarchaeota and Thermoplasmata that were well represented in our data set remained rare in MG and with no representative in SILVA (Fig. 2). These results indicate that metabarcoding data retrieved with improved primers may enable to capture a full range of archaeal diversity.

In conclusion, our newly developed primers indicate high phylogenetic diversity of Archaea in the environment that has been largely overlooked in metabarcoding studies owing to the limitations of available primers to amplify particular archaeal lineages. This is in line with growing evidence suggesting the importance of Archaea in global biogeochemical cycling (Offre *et al*., 2013). The primers developed in this study can also serve to better understand the diversity of Archaea in other habitats where they are underrepresented compared to bacteria, but functionally important, such as Methanobrevibacter in the gut (Hansen *et al*., 2011).

## Conflict of Interest

The authors declare no conflict of interest.

## Supporting information


**Fig. S1.** Rarefaction curves demonstrating the richness of detected archaeal OTUs with increasing sequencing depth using various primer pairs.

**Fig. S2.** NMDS plot showing differences in microbial phylum communities captured by the primer pairs used in this study.

**Fig. S3.** Distribution of sequence identity values between archaeal OTUs from this study and those from SILVA and MG data sets. The areas between dash lines indicate potential new archaeal taxa at various taxonomic levels (species, genera, families, orders, classes and phyla at 97%, 95%, 92%, 89%, 86% and 83% respectively; [23]), uncovered in this study. For more details, see Supporting Information Table S4.

**Table S1.** Samples used in this study.

**Table S2.** Primer pairs designed and/or tested in this study.

**Table S3.** Tag combinations and PCR conditions used for each primer.

**Table S4.** Results of *in silico* analysis of specificity and coverage for the primer pairs tested in this study.

**Table S5.** Results of Blast searches of Archaea OTU representative sequences uncovered in this study against those in SILVA and MG data sets.

**Table S6.** Comparison of Archaea recovered from this study to those in SILVA and MG data sets. The table represents a comparison of the composition of Archaea OTUs grouped into phyla and classes across the three data sets.

## Data Availability

All data are available in Supporting Information and the Short Read archive (SRA) under Accession No. SRP148434.
