# Supplementary figures and images for: Newly designed 16S rRNA metabarcoding primers amplify diverse and novel archaeal taxa from the environment
Source: Environ Microbiol Rep. 2018 Sep 12;11(4):487–94. doi: 10.1111/1758-2229.12684 (PMC6618113; doi:10.1111/1758-2229.12684)

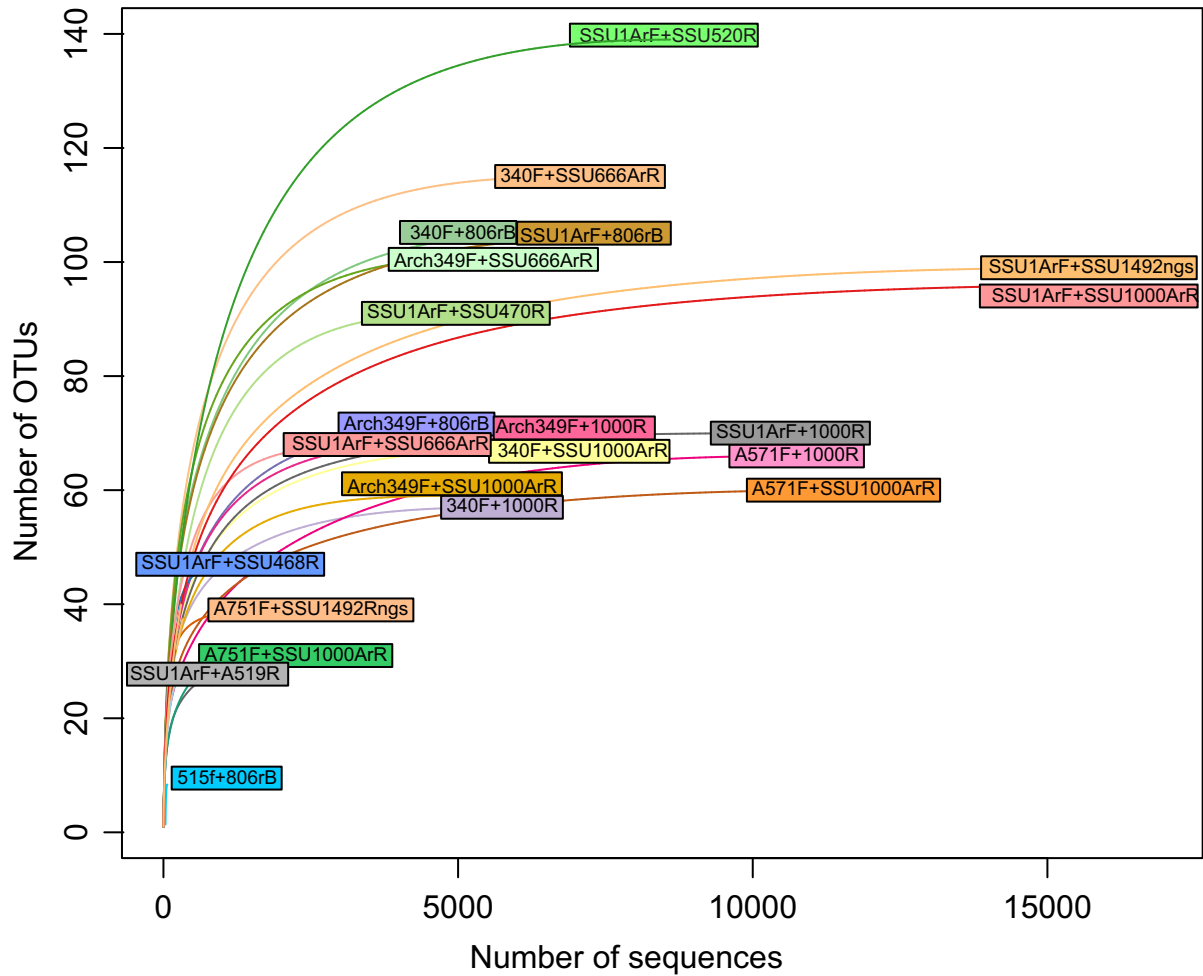

Supplement: Supplementary file 1 — Fig. S1. Rarefaction curves demonstrating the richness of detected archaeal OTUs with increasing sequencing depth using various primer pairs. [file EMI4-11-487-s001.pdf]

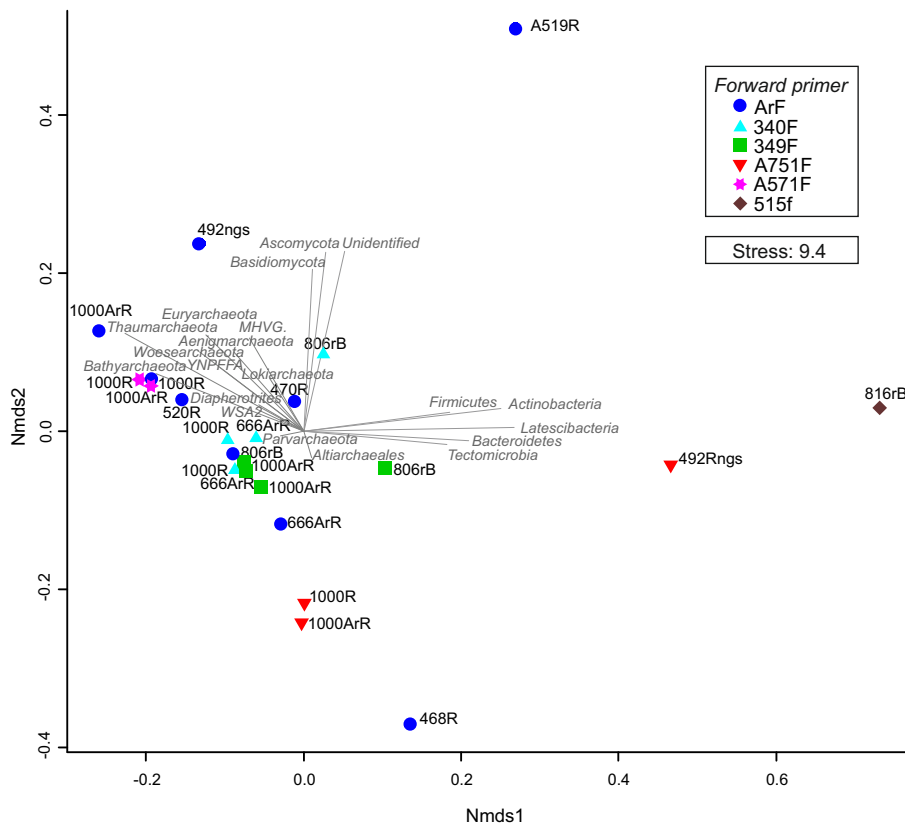

Supplement: Supplementary file 2 — Fig. S2. NMDS plot showing differences in microbial phylum communities captured by the primer pairs used in this study. [file EMI4-11-487-s002.pdf]

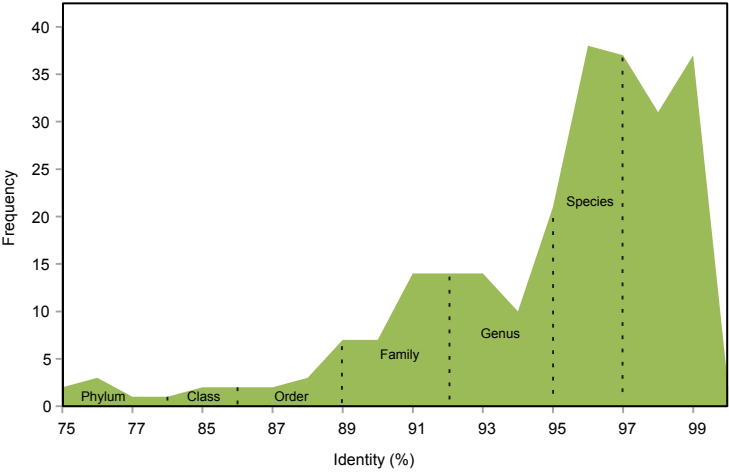

Supplement: Supplementary file 3 — Fig. S3. Distribution of sequence identity values between archaeal OTUs from this study and those from SILVA and MG data sets. The areas between dash lines indicate potential new archaeal taxa at various taxonomic levels (species, genera, families, orders, classes and phyla at 97%, 95%, 92%, 89%, 86% and 83% respectively; [23]), uncovered in this study. For more details, see Supporting Information Table S4. [file EMI4-11-487-s003.pdf]
